# Omalizumab for Idiopathic Nonhistaminergic Angioedema: Evidence for Efficacy in 2 Patients

**DOI:** 10.1155/2018/8067610

**Published:** 2018-07-22

**Authors:** Enrico Brunetta, Dana Shiffer, Marco Folci, Maria I. S. Achenza, Francesca Puggioni, Enrico Heffler, Raffaello Furlan, Giorgio W. Canonica

**Affiliations:** ^1^Department of Biomedical Sciences, Humanitas University, Milan, Italy; ^2^Internal Medicine, Humanitas Research Hospital, Milan, Italy; ^3^Personalized Medicine, Allergy and Asthma, Humanitas Research Hospital, Milan, Italy

## Abstract

Presently, there is inconclusive evidence regarding the most effective treatment for idiopathic nonhistaminergic acquired angioedema (InH-AAE). Omalizumab may, however, prove to be a promising option. This case report describes two patients who presented with recurrent angioedema attacks, which was refractory to antihistamine therapy. Hence, they were treated with 300 mg omalizumab, every 4 weeks, for a period of 6 months. Both patients had shown a rapid response to the treatment and achieved complete resolution of symptoms without further AE attacks throughout the entire duration of the treatment period. After omalizumab's suspension, one patient remained symptom free for the following 6 months and the other patient had recurrence of symptoms after 2 months for which he was retreated with omalizumab and once again became symptom free. Although omalizumab seems to be effective as a prophylactic treatment for InH-AAE, the determining factors leading to the differences in time-to-relapse between patients after its suspension remain unclear. Further studies are needed in order to better determine the potential therapeutic application of omalizumab and its role in maintenance therapy.

## 1. Introduction

Idiopathic nonhistaminergic acquired angioedema (InH-AAE) accounts for 10% of acquired AE (AAE) cases. It is defined as a nonfamilial, nonhereditary AE where other known causes of AE have been excluded and is refractory to antihistamine treatment [1]. It is predominantly seen in males, tends to occur between the ages of 36-42 years, and is associated with high recurrence rates [2, 3]. Presently, the underlying pathophysiological mechanisms in InH-AAE remain unclear. Current treatment options include bradykinin receptor antagonist icatibant, ecallantide, C1-esterase inhibitor (C1-INH) concentrate, progestin, and antifibrinolytic agent tranexamic acid (TA) [4–17]. Few cases have been described about the efficacy of dapsone, fresh frozen plasma, rituximab, and cannabis [18–21]. Omalizumab, an anti-IgE monoclonal antibody, is the first biological agent currently licensed for the treatment of chronic spontaneous urticarial (CSU) refractory to antihistamine therapy [22]. Since histamine and other mediators of activated mast cells are involved in angioedema, InH-AAE could share similarities with patients who have CSU, which do not respond to antihistamines. Recently a few case reports have shown that omalizumab was associated with positive results in patients with InH-AAE; however, data on its use in the disorder is still limited [4, 23–28].

## 2. Case Report

This case report describes 2 patients suffering from chronic recurring AE. For data use, an informed consent was obtained from both patients. The first patient, a 30-year-old Caucasian man, had a 6-year history of recurrent AE. Episodes lasted 48 hours, recurred at least twice weekly, and were characterized by swelling of the lips, eyelids, and face. The second patient, a 49-year-old Caucasian man, had a history of weekly recurring AE attacks for more than 6 months, involving the limbs and face, with each attack lasting 2-3 days.

In both patients, episodes were not trigger related; there was no history of atopy, drug/food allergy, or family history of AE. Neither the patient had taken angiotensin-converting enzyme inhibitors or gliptins. Wheals, upper airway involvement, and abdominal pain were absent during attacks. The patients had no response to H1-antihistamine treatment at 4 times the recommended daily dose. Physical examinations were unremarkable with no findings suggesting the presence of rheumatologic or autoimmune disorders.

For both patients no abnormalities were found on laboratory tests, which included complete blood count, serum protein electrophoresis/immunofixation, complement (C3/C4) levels, IgE levels, C1q, C1 esterase inhibitor levels, and function and thyroid stimulating hormone levels. ANA, ENA, anti-thyroid peroxidase antibody, and anti-thyroglobulin antibody were absent. Autologous serum skin test was positive in the first patient.

**Figure 1:**
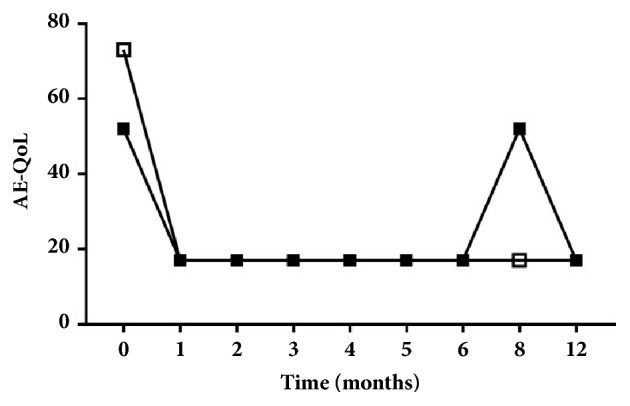
Time course of omalizumab's effect on AE-QoL score in the two patients (clear and black squares) with InH-AAE. Both patients showed a rapid response to omalizumab and during the 6-month treatment the patients were free of AE attacks. Two months after suspension one patient (black squares) had symptom recurrences and therefore was retreated with omalizumab.

The patients were treated with 300 mg omalizumab administered subcutaneously every 4 weeks for 6 months, following the conventional CSU protocol. In the first patient, clinical improvement was seen after the first omalizumab dose with the AE quality of life score (AE-QoL) decreasing from 73 to 17 [29]. He remained AE attack free during the entire treatment period and for 6 months after treatment suspension (Figure 1). In the second patient, the AE-QoL decreased from 52 to 17 a week following the first omalizumab dose. During the treatment period he was AE attack free with complete symptom resolution. Two months after treatment suspension, the AE attacks recurred and treatment was resumed. At the 4 months follow-up visit he was symptom free (Figure 1).

## 3. Discussion

In both patients, the beneficial effects from the omalizumab treatment were seen soon after the first dose was administered and ultimately resulted in complete symptom resolution. Although TA and C1-INH concentrates seem to be effective as prophylaxis treatments for InH-AAE, they are associated with lower rates of complete response compared to omalizumab [4].

Omalizumab's exact mechanism of action in InH-AAE is unclear. It is known to prevent circulating IgE from binding to high affinity immunoglobulin E receptors (Fc*ε*RI) and causes downregulation of Fc*ε*RI receptors on basophils and mast cells in AH refractory CSU cases [30]. A recent case report demonstrated a marked reduction in the density of Fc*ε*RI on basophils following omalizumab administration. It was therefore proposed that activated mast cells may potentially induce AE, by yet other unknown mediators, which is nonhistamine related [4]. Omalizumab efficacy leads to considering whether InH-AAE could be a CSU variant without manifestation of wheals.

Interestingly, the patients responded differently after omalizumab suspension. One patient remained symptom free for 6 months, while the other experienced AE recurrences after 2 months. Symptom relapse seems to occur in most patients following omalizumab discontinuation [23, 24]. Thus, long-term prophylactic treatment may be required in order to prevent recurrences [2]. The determining factors leading to differences in time-to-relapse between patients after omalizumab suspension remain unclear.

Further studies should aim to investigate the potential effects of omalizumab therapy on long-term symptom remission as well as its role as a maintenance therapy in patients with InH-AAE.

## Abbreviations

AE: Angioedema
InH-AAE: Idiopathic nonhistaminergic acquired angioedema
AE-QoL: Angioedema quality of life score
C1-INH: C1-esterase inhibitor
TA: Tranexamic acid
CSU: Chronic spontaneous urticarial
Fc*ε*RI: High affinity immunoglobulin E receptors.
